# Chlorogenic Acid Plays an Important Role in Improving the Growth and Antioxidant Status and Weakening the Inflammatory Response of Largemouth Bass (*Micropterus salmoides*)

**DOI:** 10.3390/ani14192871

**Published:** 2024-10-05

**Authors:** Zetian Xia, Haifeng Mi, Mingchun Ren, Dongyu Huang, Ahmed Mohamed Aboseif, Hualiang Liang, Lu Zhang

**Affiliations:** 1 Wuxi Fisheries College, Nanjing Agricultural University, Wuxi 214081, China; xiazetian@stu.njau.edu.cn (Z.X.); renmc@ffrc.cn (M.R.); 2 Tongwei Agricultural Development Co., Ltd., Key Laboratory of Nutrition and Healthy Culture of Aquatic, Livestock and Poultry, Ministry of Agriculture and Rural Affairs, Healthy Aquaculture Key Laboratory of Sichuan Province, Chengdu 610093, China; mihf@tongwei.com; 3 Key Laboratory of Integrated Rice-Fish Farming Ecology, Ministry of Agriculture and Rural Affairs, Freshwater Fisheries Research Center, Chinese Academy of Fishery Sciences, Wuxi 214081, China; huangdongyu@ffrc.cn; 4 National Institute of Oceanography and Fisheries (NIOF), Academy of Scientific Research and Technology (ASRT), Cairo 11796, Egypt; ahmed.aboseaf@gmail.com

**Keywords:** eco-friendly alternatives, natural feed additive, growth performance, hepatic antioxidant, hepatic gene expression, largemouth bass

## Abstract

**Simple Summary:**

This study examined the effects of chlorogenic acid (CGA) on largemouth bass’s growth, immunity, and antioxidant status. Over eight weeks, fish were fed diets with varying CGA levels. In this study, CGA improved the growth performance and lowered the FCR. In addition, CGA enhanced the antioxidant capacity and regulated the expression of genes related to immune responses, thereby strengthening immunity. Overall, the addition of CGA positively impacted the health and growth of largemouth bass.

**Abstract:**

This experiment evaluated the function of chlorogenic acid (CGA) in the growth, health status, and inflammation of largemouth bass (*Micropterus salmoides*). Over eight weeks, CGA supplementation was designed at five levels: 0, 60, 120, 180, and 240 mg/kg. The 180 and 240 mg/kg CGA-supplemented groups showed significant improvements in the FBW, SGR, and WGR compared to the control group (0 mg/kg) (*p* < 0.05). All the CGA-supplemented groups exhibited a significant reduction in the FCR (*p* < 0.05), with the 180 mg/kg CGA group showing the lowest FCR. Nonetheless, there were no appreciable differences in the plasma concentrations of TP, ALT, or AST among the treatments (*p* > 0.05). Compared to the control group, the 180 mg/kg CGA group exhibited significantly lower TC and TG levels (*p* < 0.05). The ALP levels showed no significant differences from the control group (*p* > 0.05). In terms of antioxidant parameters, CGA supplementation considerably reduced the MDA content (*p* < 0.05) and increased the GSH levels, while decreasing the *CAT*, *SOD*, and *GPx* activity levels Meanwhile, CGA supplementation resulted in reduced mRNA levels of *SOD*, *CAT*, *Nrf2*, *Keap1*, and *NF-κB* compared to the control group. In contrast, the mRNA levels of *GPx*, *IL-8*, *TLR2*, and *RelA* were elevated in the liver. Our findings indicated that CGA supplementation improved the growth performance and antioxidant status and weakened the inflammatory response of largemouth bass. These findings suggest that CGA could be a valuable dietary supplement for enhancing the health and growth of this species.

## 1. Introduction

Global aquaculture is currently experiencing rapid development, with antibiotics being widely used to enhance the growth performance and nutrient digestibility in aquatic organisms [1,2,3]. However, the extensive use of antibiotics has resulted in the emergence of drug-resistant microorganisms, environmental hazards, and significant food safety concerns [4]. Therefore, it is crucial to explore and develop alternative antibiotics that can effectively improve the health and growth of aquatic organisms, thereby promoting sustainable advancements in aquaculture.

In recent years, there has been increased interest in using natural plant extracts, particularly polyphenols with antioxidant properties, in aquatic feed. Chlorogenic acid (CGA), an ester derived from cinnamic acid and quinic acid, which is also known as 5-O-caffeoylquinic acid (5-CQA), is a prevalent polyphenol found in various fruits, vegetables, Chinese herbal medicines, and coffee beans [5]. Studies have demonstrated that CGA plays a major role in enhancing the growth performance of aquatic animals. For instance, CGA supplementation at 400, 600, and 800 mg/kg led to better protease and digestive enzyme activity levels, potentially contributing to the improved growth performance of rainbow trout (*Oncorhynchus mykiss*) [6]. Furthermore, CGA supplementation at 200 mg/kg dramatically reduced the feed conversion ratio (FCR) and increased the specific growth rate (SGR) in the crucian carp (*Carassius auratus*) [7]. CGA has also demonstrated efficacy across various animal models, exhibiting anti-inflammatory properties, enhancing antioxidant enzyme activity, and influencing specific signaling pathways [8,9]. For example, CGA supplementation at 200 mg/kg increased the antioxidant enzyme activity in crucian carps (*Carassius auratus*), especially in the liver [7]. Additionally, CGA has been found to scavenge superoxide radicals more effectively than vitamin C and to exhibit comparable abilities to vitamin E [10]. The effects of CGA on aquatic organisms have been extensively studied, particularly its anti-inflammatory properties. For instance, in channel catfish (*Ictalurus punctatus*), CGA has demonstrated the ability to prevent inflammation via the nuclear factor kappa B (*NF-κB*) signaling pathway and alleviate intestinal barrier damage resulting from oxidized fish oil feeding [11]. Moreover, CGA has been shown to have hepatoprotective effects, protecting mice from damage induced by chemical injury or lipopolysaccharide exposure [12,13].

The largemouth bass (*Micropterus salmoides*), commonly known as the California perch, is a typical freshwater carnivorous warm-water fish. Due to its rapid growth, robust stress tolerance, minimal intermuscular spines, and high nutritional value, it has become a key economic species in freshwater aquaculture. However, increasing farming densities and environmental degradation have led to a decline in immune function and a rise in disease prevalence among largemouth bass, resulting in significant economic losses. Given the well-documented adverse effects of antibiotics and their subsequent ban in many regions, finding eco-friendly alternatives has become critically important. CGA is an organic compound and a significant bioactive substance known for its growth-regulating and antibacterial properties. Despite its potential, research on CGA in largemouth bass is limited. The aim of this research was to investigate the impact of CGA supplementation on juvenile largemouth bass, in order to evaluate its potential as a viable alternative in aquaculture techniques.

## 2. Materials and Methods

### 2.1. Diet Formulation 

The diets were formulated using fish meal, chicken meal, soy protein concentrate, soybean meal, rapeseed meal, blood meal, and corn gluten meal as the main protein sources, with fish oil serving as the main lipid source. Five basic feed formulations with equal nitrogen and lipid contents were prepared with different levels of CGA (0, 60, 120, 180, and 240 mg/kg) (Table 1). All the raw materials were ground into a powder using 80-mesh screens. Fish oil and water were then thoroughly mixed with the powdered ingredients. The resulting mixture was processed into 1.5 mm pellets using an aquatic feed extruder (TSE65, Beijing, China). After extrusion, the pellets were air-dried and stored at −20 °C until needed.

### 2.2. Feeding Procedure

The entire experiment was cultured in outdoor cages at Nanjing Agricultural University’s Wuxi Fishery College. Before the main trial, the juvenile largemouth bass were acclimated in floating cages (2 × 2 × 1 m) for two weeks, receiving commercial feed to adapt to the experimental conditions. Following acclimation, 300 juveniles (3.25 ± 0.03 g) were distributed among 15 floating cages (1 × 1 × 1 m), with each cage containing 20 fish. Each level of CGA supplementation was replicated three times. Throughout the eight-week experimental period, the fish were fed twice daily, once at 7:30 AM and again at 5:30 PM, until satiation. The weights and mortality rates of the fish were recorded. The pH of the culture cycle was kept at 7.0–7.8, the dissolved oxygen content in the cages was greater than 6 mg/L, and the water temperature was 25–29 °C during the experiment.

### 2.3. Sample Collection

At the end of the eight-week feeding period, the juvenile largemouth bass underwent a 24 h fasting period. Following this, the fish were assessed for quantity and weight, and plasma and liver samples were collected from three fish per cage. Blood samples were collected through caudal venipuncture and centrifuged at 1006.2× *g* for 10 min at 4 °C to isolate the plasma layer. Liver tissues were dissected and extracted, with all the plasma and liver samples stored at −80 °C. Liver RNA was extracted using TRIzol (Nanjing Vazyme BioTech Co., Ltd., Nanjing, China). The tissue was homogenized in TRIzol, 1/5 volume of chloroform was added, and then the sample was vigorously shaken and left for 5 min. After centrifugation, the supernatant was collected, and an equal amount of isopropyl alcohol was added. Following another round of centrifugation, the supernatant was discarded, and the pellet was washed and precipitated with 75% ethanol. After centrifugation, the supernatant was discarded again and the remaining sample was precipitated, dried, dissolved in DEPC water, and transferred to the refrigerator at −80 °C. Additionally, three fish from each cage were preserved at −20 °C for a whole-body composition analysis.

### 2.4. Laboratory Trial Analysis

The AOAC method was adopted to analyze the feed and whole-body composition [14]. The blood biochemical parameters, including the total cholesterol (TC), aspartate aminotransferase (AST), alanine transaminase (ALT), glucose (GLU), alkaline phosphatase (ALP), albumin (ALB), and triglycerides (TG), were measured using the Mindray BS-400 automatic biochemical analyzer (Shenzhen, China). The intestinal malondialdehyde (MDA) levels and antioxidant markers, including superoxide dismutase (*SOD*), the total antioxidant capacity (T-AOC), catalase (*CAT*), glutathione (GSH), and glutathione peroxidase (*GPx*), were measured using test kits from the Jian Cheng Bioengineering Institute (Nanjing, China). 

### 2.5. Real-Time Quantitative PCR Analysis

A fluorescence analysis quantified the gene expression related to inflammation, immunity, and antioxidants in the livers of the fish. The gene expression was measured using StepOnePlus real-time PCR, following the analytical methodology outlined in a previous study [15]. Table 2 provides the primer sequences utilized in the analysis. Beta-actin (*β-actin*) was chosen as the stable reference gene due to its expression levels remaining unaffected by dietary factors [16]. Additionally, primers of *β-actin*, *GPx*, *CAT*, Kelch-like ECH-associated protein1 (*Keap1*), *NF-κB*, interleukin 8 (*IL-8*), and Toll-like receptor 2 (*TLR2*) were designed for this experiment.

### 2.6. Data Analysis

The data analysis was conducted using a one-way ANOVA and Duncan’s multiple comparison analysis with SPSS version 26.0, and graphical representations were created using GraphPad Prism 9.0. The results are expressed as the mean ± SEM, and statistically significant differences were found when *p* < 0.05.

## 3. Results

### 3.1. Growth Performance

Table 3 summarizes the growth results. The final body weight (FBW), weight gain ratio (WGR), and specific growth rate (SGR) were significantly higher in the 180 and 240 mg/kg CGA groups compared to the control group (*p* < 0.05). Additionally, the feed conversion ratio (FCR) was notably lower in the 180 mg/kg CGA group compared to the other CGA-supplemented groups (*p* < 0.05).

### 3.2. Whole-Body Composition

Table 4 summarizes the whole-body composition results. No significant differences (*p* > 0.05) were observed in the overall body composition of the largemouth bass across the five experimental groups.

### 3.3. Plasma Parameters

Table 5 summarizes the biochemical analysis results. There were no significant variations in the levels of TP, ALT, or AST across all the treatment groups (*p* > 0.05). A trend of decreasing plasma TC and TG levels was observed with higher doses of CGA. Specifically, the TC in the 180 mg/kg CGA group was considerably lower than that in the control group, and the TG levels also showed a significant decrease in this group (*p* < 0.05). The ALB levels were lowest at 120 and 180 mg/kg of CGA supplementation, although there were no significant differences between these levels and those of the control group (*p* > 0.05). However, the 180 mg/kg CGA group exhibited the highest GLU levels, significantly differing from the control group (*p* > 0.05).

### 3.4. Hepatic Antioxidant Parameters

**Figure 1:**
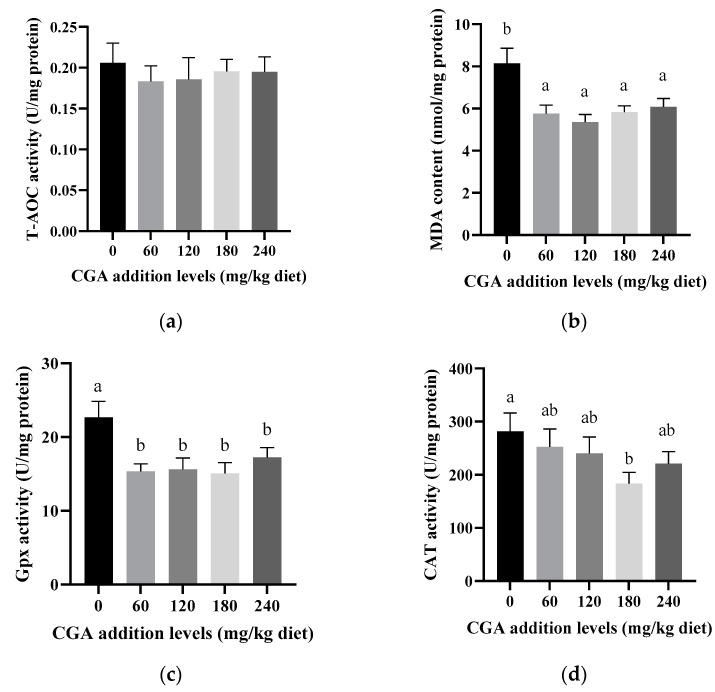

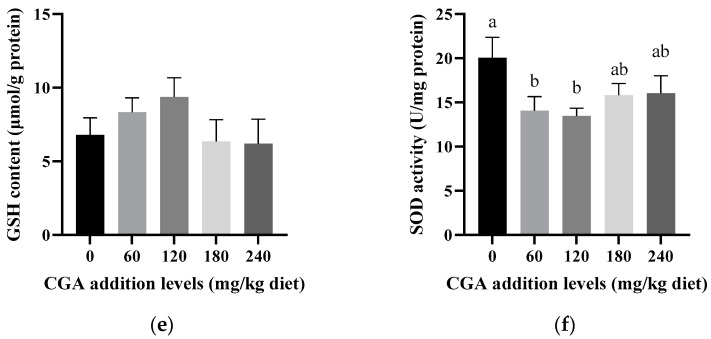
The effects of CGA on antioxidant parameters and MDA in the liver: (**a**) T-AOC; (**b**) MDA; (**c**) *Gpx*; (**d**) *CAT*; (**e**) GSH; (**f**) *SOD*. Bars marked with different letters are significantly different from each other (*p* < 0.05).

The liver’s malondialdehyde (MDA) and antioxidant parameter data are shown in Figure 1. Supplementation with 120 mg/kg of CGA significantly reduced the MDA levels compared to the control group (*p* < 0.05) (Figure 1b). At 180 mg/kg of CGA, the *CAT* activity was decreased substantially and was noticeably less than that observed in the control group (*p* < 0.05) (Figure 1d). All the CGA-supplemented groups exhibited a significant reduction in the *GPx* activity compared to the control group (*p* < 0.05) (Figure 1c). The fish in the 120 mg/kg CGA group exhibited significantly lower *SOD* activity (*p* < 0.05) (Figure 1e). However, the GSH and T-AOC levels did not show significant differences among any of the CGA treatment groups (*p* > 0.05) (Figure 1a).

### 3.5. Hepatic Gene Expression

**Figure 2:**
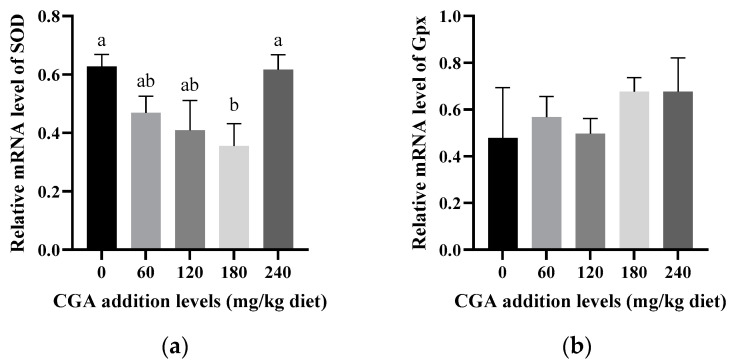

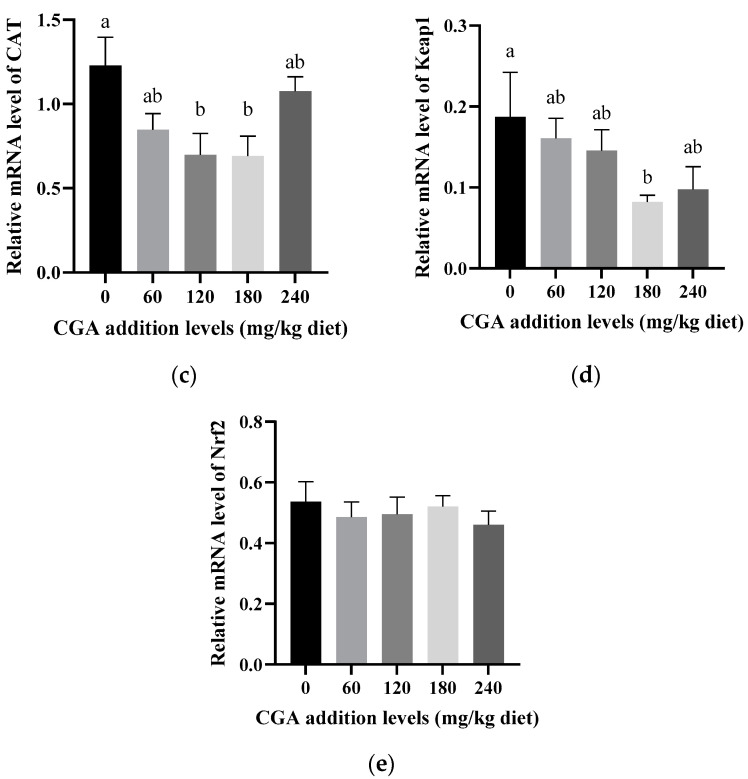
The effects of CGA on the mRNA levels of *Nrf2* signaling pathway in the liver: (**a**) *SOD*; (**b**) *Gpx*; (**c**) *CAT*; (**d**) *Keap1*; (**e**) *Nrf2*. Bars marked with different letters are significantly different from each other (*p* < 0.05).

**Figure 3:**
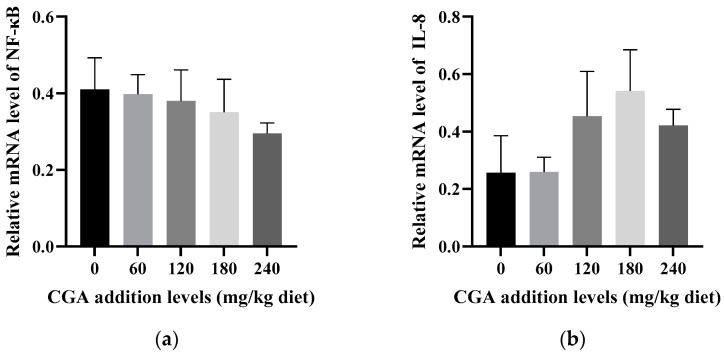

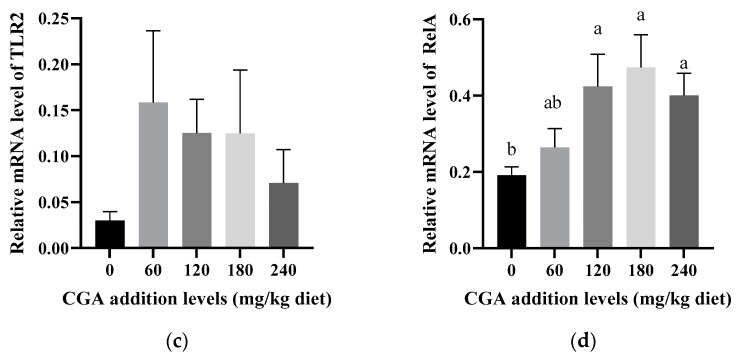
The effects of CGA on the mRNA levels of inflammatory factors: (**a**) *NF-κB*; (**b**) *IL-8*; (**c**) *TLR2*; (**d**) *RelA*. Bars marked with different letters are significantly different from each other (*p* < 0.05).

As shown in Figure 2, fish supplemented with 180 mg/kg of CGA exhibited significantly lower mRNA levels of *CAT* and *SOD* compared to the non-supplemented group (*p* < 0.05) (Figure 2a,c). Even at elevated levels of CGA, the mRNA levels of *GPx* did not differ significantly across the groups (*p* > 0.05) (Figure 2b). Similarly, the mRNA levels of *Nrf2* did not exhibit a significant decrease in comparison to the control group, regardless of the CGA concentration (*p* > 0.05) (Figure 2e). However, the mRNA levels of *Keap1* decreased significantly with increasing CGA levels up to 180 mg/kg (*p* < 0.05) (Figure 2d), before exhibiting a non-significant increase (*p* > 0.05). The mRNA levels of *NF-κB* did not show significant differences across groups (*p* > 0.05) (Figure 3a). Furthermore, the mRNA levels of *TLR2* and *IL-8* showed a pattern of increase followed by a decrease, with no significant inter-group differences (*p* > 0.05) (Figure 3c,b). Notably, the mRNA levels of *RelA* were significantly elevated in the 180 mg/kg CGA group compared to the control group (*p* < 0.05) (Figure 3d).

## 4. Discussion

CGA supplementation in feed has been reported to improve the growth performance of aquatic animals [6,7]. This study similarly observed improved growth metrics in largemouth bass, as evidenced by an increased FBW, WGR, and SGR, along with a reduced FCR. Previous research supports these findings; for instance, supplementation with 200 mg/kg of CGA significantly increased the WGR and SGR and promoted the growth of crucian carps [7]. Meanwhile, Wang et al. discovered that 200 and 400 mg/kg of CGA significantly enhanced *L. vannamei*’s resistance to the combined stress of low salinity and nitrites, as evidenced by the noteworthy increased survival [20]. Ghafarifarsani et al. observed similar effects in rainbow trout, where dosages of 600 and 800 mg/kg of CGA led to a significant increase in the WGR and a corresponding reduction in the FCR [6]. This enhancement in growth metrics may be attributed to CGA’s potential to stimulate central nervous system excitation, improve gastrointestinal contraction function, and enhance nutrient uptake and utilization [21]. Additionally, CGA might protect the liver and bile, and it exhibits antibacterial, anti-inflammatory, and immune-regulating properties [22,23,24], contributing to overall animal health and growth [25,26].

In contrast, the inclusion of CGA in this study had no discernible effect on the whole-body composition. This contrasts with the findings of Sun et al., who reported a significant reduction in the muscle crude fat content of grass carp when the CGA concentration in their feed exceeded 200 mg/kg [27]. These differences might be attributed to differences in species, feeding behavior, or environmental factors. Plasma biochemical markers are crucial indicators of metabolic state and overall health. ALT and AST serve as key biological markers for liver function tests in fish [28]. Elevated levels of these enzymes are associated with liver impairment and potential liver disease [29]. Research has demonstrated that CGA can exert a hepatoprotective effect by regulating the homeostasis of the enterohepatic axis, including improving the structure of intestinal flora and enhancing the intestinal barrier function to significantly reduce AST and ALT in mice, thus alleviating alcoholic liver injury [30]. Similarly, our study observed that CGA supplementation decreased the plasma ALT and AST levels, suggesting that CGA doses up to 240 mg/kg might protect liver function. Consistent findings were reported in channel catfish, where CGA supplementation mitigated liver damage caused by an oxidized fish oil feed [11]. All of these results indicate that CGA has great potential to protect animal livers. The mechanism may also be that CGA inhibits inflammation and enhances the antioxidant defense system by regulating the MAPK, TLR3/4, and *NF-κB* signaling pathways in animals [31,32]. However, the hepatoprotective effect in largemouth bass needs to be validated in a broader study to determine the specific effects of CGA on largemouth bass liver health. Furthermore, this study found a regulatory effect of CGA on lipid metabolism. Notably, CGA supplementation significantly diminished the TC and TG plasma levels, underscoring its beneficial modulation and hypolipidemic potential. CGA is absorbed as a prototype in the stomach and small intestine, exerting its lipid-lowering effects upon entering the bloodstream [33,34]. Yan et al. found that CGA improved the glucose tolerance, lipid metabolism, inflammation, and composition of gut microbiota in mice [35]. CGA supplementation similarly showed a positive effect in a rat model. Shimoda et al. reported that an oral administration of CGA reduced the visceral fat and lipid content in rats [36], while De Sotillo et al. found that intravenous CGA significantly decreased the TG content in the liver and plasma of rats [37], confirming its positive regulatory effect on lipid metabolism. However, the plasma glucose levels in the 180 and 240 mg/kg CGA groups were significantly higher, suggesting that excessive supplementation may elevate the risk of hyperglycemia. In conclusion, CGA may be a potent modulator of lipid metabolism, but its effect on blood glucose levels requires more careful assessment and may need to be adjusted for species and dosage.

CGA, a phenolic acid compound that is abundant in foods, is known for its potent antioxidant properties [31]. Fish tissue, rich in polyunsaturated fatty acids, is highly susceptible to damage from free radicals. An imbalance between reactive oxygen species (ROS) generation and antioxidant availability can lead to oxidative stress [38]. MDA is a reliable marker of oxidative stress, indicating lipid peroxidation in cell membranes and reflecting cellular integrity [39]. Our experiment demonstrates that CGA possesses an antioxidant capacity, reducing oxidative stress on hepatic tissues and cell membranes, as the MDA levels in the CGA-supplemented groups were considerably lower than those in the control group. Meanwhile, the scavenging of reactive oxygen species (ROS) and the functioning of antioxidant enzymes such as *CAT*, *SOD*, *GPx*, and T-AOC in fish may represent a protective mechanism against oxidative damage [40]. Our research revealed that CGA supplementation led to lower liver antioxidant enzyme activity, such as the activity of *GPx*, *CAT*, and *SOD*, contrasting with the findings reported by Jin et al. [7], which may be due to the fact that oxidative stress in the liver is alleviated under the action of CGA and, thus, the need for the activity of these antioxidant enzymes is reduced. The antioxidant enzyme activity in aquatic animals is closely related to their mRNA abundance [41]. In our study, the reduced functions of *CAT* and *SOD* in the liver of largemouth bass aligned with decreased mRNA levels, which is consistent with observations in juvenile blunt snout bream [42]. This might be because of CGA’s strong anti-inflammatory and antioxidant capabilities, enhancing cell membrane integrity by scavenging free radicals in the liver [43]. *Keap1* is normally a negative regulatory protein of *Nrf2*, inhibiting its transcriptional activity by binding to its amino-terminal regulatory domain [44]. Other research has shown that stressful conditions or exposure to unfavorable nutrients, such as dairy cows under heat stress [45] or largemouth bass fed inappropriate starch sources [46], can lead to higher oxidative stress. In some cases, however, even in the absence of obvious oxidative stress, cells can prepare for the potential activation of *Nrf2* by cells, possibly by increasing *Keap1* expression, a preventive regulation to respond quickly to possible oxidative challenges. In addition, *Keap1* expression may be regulated by various factors, including hormone levels, the nutritional status, the cell cycle, and the differentiation status [47]. Therefore, the high level of *Keap1* mRNA in the control group may have resulted from the combined effect of these factors, and the specific mechanism needs to be further studied. As a polyphenol compound, CGA has powerful antioxidant properties, which can reduce oxidative stress and free radical damage to cells [31]. It is possible that CGA supplementation through its antioxidant effect reduces the oxidative stress in cells and, thus, reduces the level of *Keap1* mRNA. This is consistent with the finding that the mRNA levels of *SOD* and *CAT* were decreased, though *Nrf2* expression itself did not show significant changes. This might be because *Nrf2* protein levels are tightly regulated by *Keap1*, and rapidly degrade under normal conditions, but stabilize under stress conditions [48,49]. Furthermore, CGA supplementation resulted in the decreased activity of antioxidant enzymes (e.g., *GPx*, *CAT*, and *SOD*) in the liver, probably because CGA reduces oxidative and inflammatory stress by regulating the expression of antioxidant and cytoprotective genes. Although there is no direct evidence on how CGA affects *Keap1*′s sensitive cysteine residue modification, it is reasonable to speculate that CGA may influence the *Keap1* function directly or indirectly, thereby affecting *Nrf2* activity and the antioxidant response of cells. Future studies should further investigate the specific mechanisms of the interaction between CGA and *Keap1*.

Tissue damage in fish can lead to inflammation, which is often characterized by the elevated mRNA expression of cytokines [50]. *NF-κB* is a crucial transcription factor involved in inflammatory responses [51]. In this study, the mRNA levels of *NF-κB* tended to decline with increasing doses of CGA supplementation, although they did not change significantly. These findings suggest that CGA might suppress the increase in the *NF-κB* abundance, potentially mitigating the hepatic inflammatory response by preventing the activation of the *NF-κB* signaling pathway. Several research works have demonstrated the anti-inflammatory characteristics of CGA [9,12,52]. Additionally, the mRNA levels of *RelA*, *TLR2*, and *IL-8* initially increased and then decreased, with significant elevations in the *RelA* mRNA levels observed at CGA supplementation levels of 120–240 mg/kg. This suggests that CGA may have an effect on the immune status of largemouth bass at specific doses by modulating the signaling pathways related to immune responses. Specifically, CGA may affect the maturation and activation of immune cells, as well as the production of inflammatory factors, through the activation of pattern recognition receptors (e.g., TLR 2) and transcription factors (e.g., *RelA*, a component of *NF-κB*). Despite the observed effects, CGA had no significant impact on *NF-κB*, indicating that CGA might regulate inflammatory responses through mechanisms beyond the *NF-κB* signaling pathway. This is consistent with other findings, such as CGA’s ability to ameliorate inflammatory liver damage by inhibiting HSP60-mediated inflammatory signaling pathways [53]. However, there is a lack of research on the specific regulatory mechanisms of CGA on inflammatory factors, and further investigation is needed to elucidate these mechanisms.

## 5. Conclusions

Adding CGA to the diet of largemouth bass can improve their growth and antioxidant status and weaken their inflammatory response, while also regulating their lipid metabolism and showing potential to protect their liver. As a natural feed additive, CGA is expected to be an effective alternative to antibiotics in aquaculture due to its multifaceted benefits.

## Figures and Tables

**Table 1:** Experimental basic formula (% dry matter).

Ingredients (g/kg)	Level (%)	Ingredients (g/kg)	Level (%)
Fish meal ^1^	30.00	Fish oil	6.66
Chicken meal ^1^	5.00	Cassava starch	5.00
Soy concentrated protein ^1^	8.00	Choline chloride	0.50
Soybean meal ^1^	11.00	Vitamin premix ^2^	1.00
Rapeseed meal ^1^	7.20	Mineral premix ^2^	1.00
Blood meal ^1^	5.00	Monocalcium phosphate	3.46
Corn gluten meal ^1^	7.70	Vitamin C	0.05
Wheat gluten ^1^	3.00	L-lysine ^3^	0.28
Wheat meal	5.00	L-methionine ^3^	0.15
Analyzed proximate composition (dry matter)			
Crude protein (%)	47.50 ± 0.08		
Crude lipid (%)	10.86 ± 0.08		
Gross energy (KJ/g)	20.23 ± 0.23		

Note: ^1^ The crude protein contents of fish meal, chicken meal, soy concentrated protein, soybean meal, rapeseed meal, blood meal, corn gluten meal, and wheat gluten were 65.80%, 62.00%, 63.00%, 46.00%, 39.00%, 90.00%, 60.57%, and 80.00%, and the crude lipid contents were 9.50%, 9.00%, 4.10%, 4.25%, 6.00%, 0.00%, 3.30%, and 2.00%, respectively. Biomar Tongwei Biotech Co., Ltd. (Wuxi, China) provided them. ^2^ Hanove Animal Health Products Co., Ltd. (Wuxi, China) provided the vitamin premix and mineral premix. ^3^ Feeer Co., Ltd. (Shanghai, China) provided L-lysine and L-methionine.

**Table 2:** Real-time PCR primer sequences.

Target Gene	Forward (5′-3′)	Reverse (5′-3′)	GenBank
*β-actin*	GGTGTGATGGTTGGTATGG	CTCGTTGTAGAAGGTGTGAT	MH018565.1
*GPx*	GAAGGTGGATGTGAATGGA	CCAACCAGGAACTTCTCAA	MK614713.1
*SOD*	TGGCAAGAACAAGAACCACA	CCTCTGATTTCTCCTGTCACC	[17]
*CAT*	CTATGGCTCTCACACCTTC	TCCTCTACTGGCAGATTCT	MK614708.1
*Nrf2*	CCACACGTGACTCTGATTTCTC	TCCTCCATGACCTTGAAGCAT	[18]
*Keap1*	CGTACGTCCAGGCCTTACTC	TGACGGAAATAACCCCCTGC	XP_018520553.1
*NF-κB*	CCACTCAGGTGTTGGAGCTT	TCCAGAGCACGACACACTTC	XP_027136364.
*RelA*	GCTGGTGTCTGGTTCATT	GCCTCCTCTTCCATCTCT	[19]
*IL-8*	GAGGGTACATGTCTGGGGGA	CCTTGAAGGTTTGTTCTTCATCGT	XM_038713529.1
*TLR2*	TCGCTGTTCACCAATCTG	TAGTTCTCCTCTCCATCTGT	MN807054

Note: *GPx*, glutathione peroxidase; *Nrf2*, nuclear factor erythroid 2-related factor 2; *RelA*, V-rel avian reticuloendotheliosis viral oncogene homolog A; *SOD*, superoxide dismutase; *CAT*, catalase; *IL-8*, interleukin 8; *Keap1*, Kelch-like ECH-associated protein1; *NF-κB*, nuclear factor kappa B; *TLR2*, Toll-like receptor 2.

**Table 3:** Effects of CGA on the growth performance (mean ± SEM).

Parameters	CGA Addition Levels				
	0 mg/kg	60 mg/kg	120 mg/kg	180 mg/kg	240 mg/kg
IBW (g)	3.25 ± 0.01	3.24 ± 0.01	3.24 ± 0.01	3.24 ± 0.01	3.26 ± 0.01
FBW (g)	21.82 ± 2.40 ^b^	25.26 ± 0.37 ^ab^	25.80 ± 1.50 ^ab^	28.10 ± 1.18 ^a^	27.60 ± 1.05 ^a^
WGR (%)	570.38 ± 75.15 ^b^	680.34 ± 12.52 ^ab^	700.29 ± 49.01 ^ab^	767.96 ± 36.88 ^a^	746.53 ± 32.95 ^a^
SGR (%/day)	3.39 ± 0.20 ^b^	3.67 ± 0.03 ^ab^	3.71 ± 0.11 ^ab^	3.86 ± 0.08 ^a^	3.81 ± 0.07 ^a^
FCR	1.46 ± 0.07 ^a^	1.31 ± 0.01 ^b^	1.20 ± 0.02 ^bc^	1.16 ± 0.02 ^c^	1.18 ± 0.05 ^c^

Note: Data are expressed as mean ± SEM. Statistical significance of differences is indicated by distinct letters (a, b, c) on same line (*p* < 0.05). IBW, initial body weight. WGR (%) = × 100 (W_2_ (g) − W_1_ (g))/W1 (g). SGR (%/d) = 100 × [(Ln (W_2_ (g)) − Ln (W_1_ (g)))/days]. FCR = W_3_ (g)/W_4_ (g), where W_1_ is initial body weight, W_2_ is final body weight, W_3_ is dry feed fed, and W_4_ is wet weight gain.

**Table 4:** Effects of CGA on whole-body composition.

Parameters	CGA Addition Levels				
	0 mg/kg	60 mg/kg	120 mg/kg	180 mg/kg	240 mg/kg
Moisture (%)	72.47 ± 0.22	72.32 ± 0.10	72.48 ± 0.27	72.18 ± 0.03	72.41 ± 0.12
Crude protein (%)	16.30 ± 0.21	16.95 ± 0.30	16.61 ± 0.09	17.06 ± 0.37	17.03 ± 0.44
Crude lipid (%)	7.48 ± 0.38	6.87 ± 0.51	7.40 ± 0.30	7.06 ± 0.19	7.31 ± 0.58
Ash (%)	3.80 ± 0.07	3.59 ± 0.02	3.86 ± 0.14	3.93 ± 0.14	3.82 ± 0.09

Note: Data are presented as mean ± SEM.

**Table 5:** Effects of CGA on plasma parameters.

Parameters	CGA Addition Levels				
	0 mg/kg	60 mg/kg	120 mg/kg	180 mg/kg	240 mg/kg
TC (mmol/L)	8.37 ± 0.39 ^a^	7.57 ± 0.22 ^ab^	7.53 ± 0.35 ^ab^	7.04 ± 0.30 ^b^	7.85 ± 0.38 ^ab^
TP (g/L)	33.23 ± 1.17	31.69 ± 0.98	31.59 ± 1.05	30.68 ± 1.18	33.21 ± 1.67
TG (mmol/L)	7.19 ± 0.68 ^a^	7.36 ± 0.60 ^a^	6.27 ± 0.50 ^ab^	5.37 ± 0.29 ^b^	6.84 ± 0.67 ^ab^
AST (U/L)	15.64 ± 1.14	16.80 ± 3.98	13.66 ± 3.20	12.54 ± 2.16	11.23 ± 6.41
ALT (U/L)	1.73 ± 0.45	1.46 ± 0.37	1.56 ± 0.45	1.20 ± 0.33	1.34 ± 0.52
ALB (g/L)	9.98 ± 0.41 ^ab^	9.92 ± 0.40 ^ab^	9.83 ± 0.40 ^ab^	9.90 ± 0.42 ^b^	10.46 ± 0.48 ^a^
GLU (mmol/L)	5.80 ± 0.61 ^c^	5.99 ± 0.45 ^c^	5.88 ± 0.42 ^c^	8.79 ± 0.52 ^a^	7.53 ± 0.48 ^b^

Note: Data are expressed as mean ± SEM. Statistical significance of differences is indicated by distinct letters (a, b, c) on the same line (*p* < 0.05).

## Data Availability

The data are contained within the article.
